# Modulation of the Root Microbiome by Plant Molecules: The Basis for Targeted Disease Suppression and Plant Growth Promotion

**DOI:** 10.3389/fpls.2019.01741

**Published:** 2020-01-24

**Authors:** Alberto Pascale, Silvia Proietti, Iakovos S. Pantelides, Ioannis A. Stringlis

**Affiliations:** ^1^ Department of Agricultural Sciences, University of Naples Federico II, Naples, Italy; ^2^ Department of Ecological and Biological Sciences, University of Tuscia, Viterbo, Italy; ^3^ Department of Agricultural Sciences, Biotechnology and Food Science, Cyprus University of Technology, Limassol, Cyprus; ^4^ Plant-Microbe Interactions, Department of Biology, Science4Life, Utrecht University, Utrecht, Netherlands

**Keywords:** plant defense, plant growth promotion, plant molecules, root exudation, root microbiome, microbiota, disease suppression, microbial inoculants

## Abstract

Plants host a mesmerizing diversity of microbes inside and around their roots, known as the microbiome. The microbiome is composed mostly of fungi, bacteria, oomycetes, and archaea that can be either pathogenic or beneficial for plant health and fitness. To grow healthy, plants need to surveil soil niches around the roots for the detection of pathogenic microbes, and in parallel maximize the services of beneficial microbes in nutrients uptake and growth promotion. Plants employ a palette of mechanisms to modulate their microbiome including structural modifications, the exudation of secondary metabolites and the coordinated action of different defence responses. Here, we review the current understanding on the composition and activity of the root microbiome and how different plant molecules can shape the structure of the root-associated microbial communities. Examples are given on interactions that occur in the rhizosphere between plants and soilborne fungi. We also present some well-established examples of microbiome harnessing to highlight how plants can maximize their fitness by selecting their microbiome. Understanding how plants manipulate their microbiome can aid in the design of next-generation microbial inoculants for targeted disease suppression and enhanced plant growth.

## Introduction

Plants are sessile organisms anchored in the soil by their roots. In terrestrial ecosystems, plants are the main food producers supporting most of the other life. In nature, plants are continuously exposed to various biotic stresses caused by pathogens or pests and adverse environmental conditions, such as drought, soil salinity, extreme temperatures, nutrient deficiencies, or exposure to heavy metals (De Coninck et al., 2015; Antoniou et al., 2017; Hacquard et al., 2017). To survive biotic stresses, plants have evolved an array of sophisticated immune responses which protect plant cells from the challenges they confront (Pieterse et al., 2012; Pieterse et al., 2014). For decades, the interactions between plants and pathogens were studied under the prism of an individual plant–microbe relationship, ignoring the complexity of such interactions and the involvement of many other groups of microorganisms that affect the outcome of infection (Mendes et al., 2011; Berendsen et al., 2012; Bulgarelli et al., 2013). Over the last years, focus has been diverted to the effect of the plant-associated microbial communities on plant growth and health. Increasing evidence suggests that services provided by plant-associated microorganisms can broaden immune functions of the plant host (Vannier et al., 2019). It has even been postulated that plants actively recruit soil microorganisms by releasing compounds in the rhizosphere that selectively stimulate microorganisms that are beneficial to plant growth and health (Reinhold-Hurek et al., 2015; Sasse et al., 2017). Here, we review the current understanding on the composition and activity of the root-associated microbial communities, and we discuss how different plant molecules can shape the structure of these communities providing also with examples on the interactions between plants and soilborne fungi.

### Plants and Microbiome

#### Game of Biomes: Plants Roots and Their Microbiome

**Figure 1:**
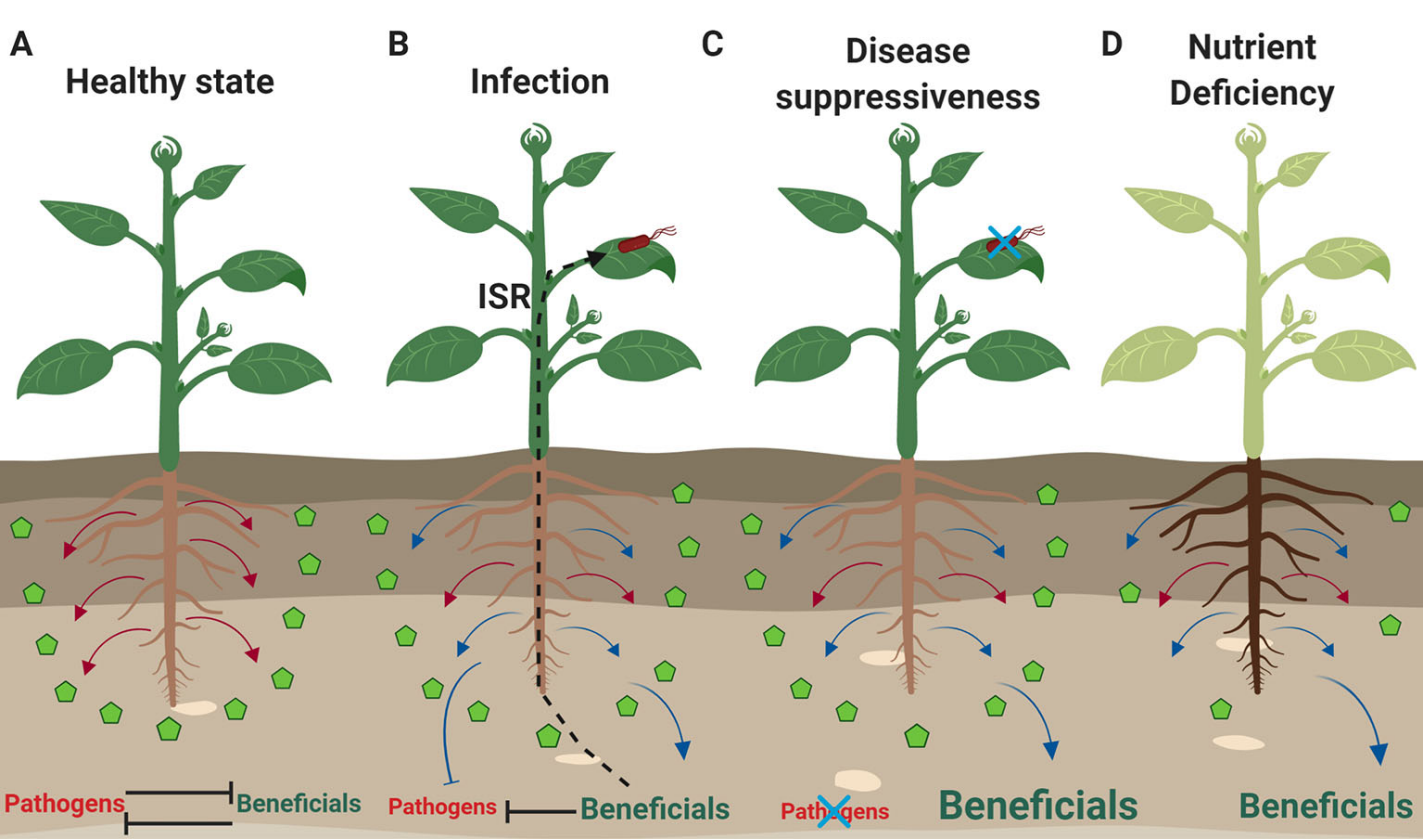
Plants respond to different environmental stresses and modulate their microbiome. **(A)** Plants not experiencing any biotic stress and having access to nutrients (green pentagons), release constitutively exudates (red arrows) that allow them to sustain a balance in the rhizosphere between pathogenic and beneficial microbes. **(B)** Upon infection by a pathogen (red microbe), the exudation profile of roots changes and stress-induced exudates (blue arrows) aid the plants in inhibiting pathogenic growth in the rhizosphere, while selecting at the same time for beneficial microbes. Some of these beneficial microbes when they establish themselves in the rhizosphere, can trigger ISR that can help plants deal with pathogenic infections in the leaves. **(C)** In the case of soil suppressiveness or “cry-for-help” conditions, there is establishment of beneficial rhizosphere communities that are further supported by the release of stress-induced exudates. Under these conditions, soilborne and foliar pathogens fail to cause disease. **(D)** Plants experiencing nutrient deficiencies (e.g. iron, nitrogen, phosphate) change the metabolomic profile of their roots to either make nutrients more available and soluble or to attract beneficial microbes (e.g. rhizobia, AMF, PGPR) that can help them deal with the nutrient deficiency. Font size indicates the abundance of beneficial or pathogenic subsets of the microbiota under different conditions. The figure was designed with Biorender (https://biorender.com).

Plants harbor a mesmerizing diversity of microbes both in their aboveground and their belowground tissues that are collectively known as plant microbiota, while the genomes of the microbiota living in close association with plants are commonly referred to as the plant microbiome (Berendsen et al., 2012; Bulgarelli et al., 2013). This review will focus on the interactions of the microbiome with the root, which is the plant organ “hidden” in the soil that mediates key functions for plant longevity and fitness (De Coninck et al., 2015). Some of these functions are the fixation of a plant in a position, the uptake and storage of nutrients and water from the soil and the mediation of the interaction with soil-inhabiting microbes (**Figure 1**). Roots and their surrounding soil constitute one of the most rich and diverse ecosystems on Earth. The grand concentration of microbial life in the thin soil layer surrounding the roots, known as the rhizosphere, is explained by the release of carbon-rich products of photosynthesis which are a vital food source for the attracted microbes (Bais et al., 2006; Sasse et al., 2017). Rhizodeposits are quite diverse and include organic acids, amino acids, sugars, products of secondary metabolism, and even the release of dying root cap border cells (Dakora and Phillips, 2002; Bais et al., 2006; Driouich et al., 2013). Root-derived exudates, apart from supporting microbial proliferation in the rhizosphere, are also responsible for the formation of distinct microbial assemblages between soil and the rhizosphere, a phenomenon described as the “rhizosphere effect” (Hiltner, 1904; Berendsen et al., 2012). The microbes proliferating in the rhizosphere are therefore exposed to plant-derived compounds and signaling molecules and represent a subset of the highly complex microbial communities of the bulk soil (Berendsen et al., 2012). A next layer of selection occurs when microbes grow on the root surface (rhizoplane) or inside roots (endosphere) and in turn less diverse microbial communities are observed (Bulgarelli et al., 2013; Reinhold-Hurek et al., 2015; Hacquard et al., 2017). These layers of selection are critical considering that the root-associated microbiota consist of microbes that can assist plants in nutrient assimilation, or enhance their growth and defense potential, but also of microbes that can be detrimental for plant health (Lugtenberg and Kamilova, 2009; Pieterse et al., 2014; De Coninck et al., 2015). Therefore, the maintenance of a balance between plant health and the accommodation of this plethora of microbes in the root rhizosphere requires a coordination of complex processes in the rhizosphere where all partners benefit (Zamioudis and Pieterse, 2012).

#### The Identity of Root-Associated Microbiomes

The last decade several studies unearthed the composition of root-associated microbial communities. Most of these studies employed next-generation sequencing of microbial marker genes like 16S rRNA for bacteria and the nuclear ribosomal internal transcribed spacer (ITS) region for fungi (Claesson et al., 2010; Schoch et al., 2012) which is known as amplicon sequencing (Sharpton, 2014), while others used shotgun metagenomics sequencing where not only selected microbial marker genes but all DNA present in an environmental sample is sequenced (Sessitsch et al., 2012; Ofek-Lalzar et al., 2014; Bai et al., 2015; Bulgarelli et al., 2015; Stringlis et al., 2018b). The latter approach allows not only for the taxonomic profiling of the root-associated microbial communities but also for the functional characterization of the microbiome (Sharpton, 2014). These culture-independent methodologies allowed the characterization of the microbiota in both the rhizosphere but also in the endosphere of different plant species. In the case of bacteria, analysis at phylum level revealed that the microbiota of healthy *Arabidopsis thaliana* (hereafter Arabidopsis) plants originates from the more diverse soil communities, and is dominated by the phyla Proteobacteria, Actinobacteria, Bacteroidetes and less by Firmicutes (Bulgarelli et al., 2012; Lundberg et al., 2012). Similarly, the root microbiome of closely related species belonging to the Brassicaceae family (*Cardamine hirsuta*, *Arabidopsis halleri*, *Arabidopsis lyrata* and *Arabis alpina*) display quite similar root microbial assemblages with those of Arabidopsis (Schlaeppi et al., 2014; Dombrowski et al., 2017). In plant species not related to Arabidopsis, such as barley, citrus, rice, *Lotus japonicus*, poplar, sugarcane, and tomato, the phyla Proteobacteria, Actinobacteria, Bacteroidetes, and Firmicutes constitute the highest proportion among the identified bacteria (Bulgarelli et al., 2015; Edwards et al., 2015; De Souza et al., 2016; Zgadzaj et al., 2016; Beckers et al., 2017; Zhang et al., 2017; Kwak et al., 2018). For fungal communities, studies in Arabidopsis, *Arabis alpina*, poplar, and sugarcane have shown that mostly the phyla Ascomycota, Basidiomycota and less Zygomycota, and Glomeromycota dominate the root microbiota of their host plants (Shakya et al., 2013; De Souza et al., 2016; Almario et al., 2017; Robbins et al., 2018; Bergelson et al., 2019). The high representation of selected bacterial and fungal phyla in roots and rhizospheres of different hosts suggests that members of these phyla constitute competitive and adaptable colonizers under various soil types and locations (Muller et al., 2016). Indeed, sequencing of microbiome DNA and RNA from the rhizosphere and the root of *Brassica napus* and citrus demonstrated that phyla Proteobacteria, Actinobacteria, Acidobacteria and Bacteroidetes are really active in the root and the rhizosphere and assimilate most of the carbon released by the roots (Gkarmiri et al., 2017; Zhang et al., 2017). Metatranscriptomics, functional studies or labelling of carbon absorption revealed that overrepresentation of specific fungal phyla in the rhizosphere correlates with their increased activity around the roots or services they provide to the host plants (Vandenkoornhuyse et al., 2007; Turner et al., 2013; Almario et al., 2017; Gonzalez et al., 2018).

#### Interactions of Plants With Beneficial and Pathogenic Microbes

##### Beneficial Associations With Plants

###### Symbiotic Plant-Microbe Associations

Research has unearthed that intimate interactions of plants with beneficial microbes first occurred millions of years ago. The first land plants were colonized by ancestral filamentous fungi that facilitated water absorption and nutrient acquisition for the host plant, while fungi received back photosynthetically-fixed carbon (Field et al., 2015; Martin et al., 2017). This symbiotic association coevolved in such a successful direction since more than 90% of living plant species form symbioses with mycorrhizal fungi, of which about 80% are classified as arbuscular mycorrhizal fungi (AMF) (Parniske, 2008; Bonfante and Genre, 2010). As obligate biotrophs, AMF need to sense the presence of the host plants to complete their lifecycle. The root-exuded plant hormone strigolactone has been recognized as the stimulatory signal for AMF mycelium metabolism and branching and its concentration gradient from the roots reveal the proximity to the host plant (Parniske, 2008; Bonfante and Genre, 2010). Intriguingly, AMF signaling pathways are very similar to the one that coordinates the well-known symbiosis between the paraphyletic group of rhizobial bacteria and leguminous plants and are therefore named common signaling symbiotic pathways (CSSPs) (Maclean et al., 2017; Martin et al., 2017). In rhizobia, the symbiotic association begins with the perception of specific root-exuded iso-flavonoid compounds by the microbes that stimulates root nodule formation (Begum et al., 2001; Oldroyd, 2013; Poole et al., 2018). Once symbiosis is established there is continuous exchange of nutrients between the host plant and the microbes. AMF can uptake the consistently low water-soluble inorganic orthophosphate (Pi) from soils and transport Pi through the extraradical mycelium network and fungal arbuscules inside the root. AMF can also uptake and transport other major nutrients; for example nitrogen is transferred in the forms of nitrate, ammonium, and amino acids inside plants by using specialized transporters (Parniske, 2008; Bonfante and Genre, 2010; Maclean et al., 2017). In exchange, AMF receive the entire carbon requirements from plants, through specific fungal hexose transporters and fatty acids (Jiang et al., 2017; Maclean et al., 2017). In rhizobia-leguminous plants symbiosis, rhizobia reduce atmospheric N_2_ to ammonia inside the root nodules and secrete it to plants, while plants provide rhizobia with dicarboxylates (Poole et al., 2018).

###### Nutrient Uptake and Growth Promotion by Beneficial Microbes

Plants can acquire nutrients even in the absence of symbiosis with AMF or rhizobia. Enhanced nutrient acquisition in plants is a very common mechanism of phytostimulation (Lugtenberg and Kamilova, 2009; Finkel et al., 2017; Jacoby et al., 2017; Verbon et al., 2017) and a wide array of microbes can accomplish this function in non-mycorrhizal plants (Almario et al., 2017; Castrillo et al., 2017; Fabianska et al., 2019). The non-host plant Arabidopsis acquires Pi through its natural root endophytic symbiont *Colletotrichum tofieldiae* (Hiruma et al., 2016). Hiruma and colleagues (2016) demonstrated that Pi translocation is the main plant growth promotion mechanism provided by *C. tofieldiae* and this mechanism is governed by the plant phosphate starvation status and requires intact immune system of the plant. Endophytic fungi belonging to the order of Sebacinales, such as *Serendipita indica* (formerly known as *Piriformospora indica*) can also promote plant growth through Pi acquisition (Yadav et al., 2010; Weiss et al., 2016). Similarly, *Trichoderma* fungi can produce chelating metabolites that solubilize phosphate and increase its acquisition by plants to promote plant growth (Altomare et al., 1999; De Jaeger et al., 2011). Nitrogen acquisition is mediated on non-leguminous plants by other microbes which are not belonging in the N-fixing bacteria group (Jacoby et al., 2017; Martin et al., 2017). Evidence also accumulates that during root colonization selected beneficial microbes can hijack the iron deficiency response of plants. In this case, following bacterial colonization there is induction of the expression of genes with a role in iron uptake, and these genes are commonly used by plants to mobilize and uptake iron, when this element is present in unavailable forms in the soil (Zamioudis et al., 2015; Zhou et al., 2016; Martinez-Medina et al., 2017; Verbon et al., 2017).

Beneficial microbes can promote plant growth by affecting the hormonal balance of plants. This beneficial effect can be induced by the secretion of microbial small secondary metabolites (SM) that can act as hormone-like plant growth regulators, or by the production of SM and proteins that enable microbes to modulate the signaling of plant defense hormones to successfully colonize plant tissues (Verbon and Liberman, 2016; Patkar and Naqvi, 2017; Manganiello et al., 2018; Stringlis et al., 2018c). Numerous microbial species among plant associated bacteria and fungi can produce indole-3-acetic acid (IAA) or auxin-mimicking molecules that play a direct role on plant growth and development (Duca et al., 2014; Garnica-Vergara et al., 2016). Other microbial phytohormones or phytohormone-like molecules, such as cytokinins, gibberellins and analogues of defense-related hormones, such as salicylic acid (SA) or jasmonic acid (JA)-isoleucine are mainly produced to facilitate microbial colonization through modulation of plant immunity (Schafer et al., 2009; Stringlis et al., 2018c). Moreover, many plant beneficial microorganisms produce 1-aminocyclopropane-1-carboxylate (ACC) deaminase that cleaves ACC, the immediate biosynthetic precursor of ethylene (ET) in plants, and promote plant growth presumably by lowering plant ET which can reach inhibitory levels for plant growth when subjected to stress conditions (Viterbo et al., 2010; Brotman et al., 2013; Glick, 2014; Stringlis et al., 2018c).

###### Induced Systemic Resistance

Another well-studied mechanism of elevated plant defense potential is the so-called induced systemic resistance (ISR) which is triggered by beneficial members of the root microbiome to a wide range of plant hosts making them resistant against various pathogenic threats (Pieterse et al., 2014). Systemic activation of plant defenses is ensured by a complex network of defense-related hormone signaling pathways, which brings the message of a beneficial interaction, in different plants organs (Pieterse et al., 2009; Pieterse et al., 2014). The ISR phenomenon has been firstly described for bacteria of the genus *Pseudomonas,* and this mechanism has been distinguished from “systemic acquired resistance” (SAR) which is induced by pathogens (Pieterse et al., 2014). ISR has also been described for many plant growth-promoting bacteria (PGPR) of the genus *Bacillus* and *Serratia* and plant growth-promoting fungi (PGPF) of the genus *Trichoderma, Fusarium, Serendipita* and AMF (Harman et al., 2004; Kloepper et al., 2004; Shoresh et al., 2010; Jung et al., 2012; Pieterse et al., 2014) and is determined by the perception of microbial secreted SM (Ongena and Jacques, 2008; Raaijmakers et al., 2010; Manganiello et al., 2018; Stringlis et al., 2018c). Interestingly, ISR is characterized by the activation of defense responses only after pathogen attack, saving the plant from a great energy consumption. This mechanism of “upon attack” defense activation is known as priming and is an energy-saving evolutionary strategy that allows plants to silently alert their immune system until a challenge by pathogens or insects occurs. Following this challenge, plants will deploy all the cellular responses faster and/or stronger resulting in a more efficient and effective resistance (Pieterse et al., 2014; Martinez-Medina et al., 2016).

All the beneficial associations presented above are based on the interaction between the host plant and a single beneficial microbe. Modern holistic approaches aim to correlate plant health to the entire plant-associated microbial community. In this case, microbial genes are considered as an extension of the plant genetic repertoire and perform specific functions benefiting plant growth, reproduction and disease resistance (Vandenkoornhuyse et al., 2015; Hassani et al., 2018). Community level-based metagenomic studies can elucidate whether there is functional redundancy or overlapping genomic traits in most microbes promoting plant growth or inducing systemic resistance, enabling in this way the discovery of novel PGPR or PGPF (Lugtenberg and Kamilova, 2009; Pieterse et al., 2014; Bai et al., 2015; Zeilinger et al., 2016; Berendsen et al., 2018; Duran et al., 2018).

##### Plant-Pathogen Interactions

During plant life, roots support beneficial associations with soil-inhabiting microbes but need to cope at the same time with the infections caused by pathogenic microorganisms. Soilborne pathogens can affect hundreds of plant species, including economically important crops, and cause significant monetary losses due to significant reduction in yield and quality. For many crops, losses are estimated at 10%–20% of the attainable yield (Pimentel et al., 1991; Okubara and Paulitz, 2005; De Coninck et al., 2015). However, crop losses are often underestimated as soilborne pathogens are not an immediate concern for growers and their practices in many cases lead to increased inoculum reservoirs in soils (Chellemi et al., 2016). Also, their economic importance is expected to significantly rise due to the increasing implementation of conservation tillage or no-till farming practices in many countries (De Coninck et al., 2015) and the climate change that can increase their geographical range on Earth (Cheng et al., 2019). Soilborne pathogens reside in the soil for short or extended periods, and survive as saprophytes on plant residues and organic matter or as resting structures (e.g. sclerotia, chlamydospores, oospores, melanized mycelia) until triggered to grow by root exudates (Bruehl, 1987; Bais et al., 2006; De Coninck et al., 2015). For example, phenolic acids, sugars, and free amino acids in root exudates from watermelon significantly increased spore germination and sporulation of *F. oxysporum* f. sp. *niveum* (Hao et al., 2010). Similarly, tomato root exudates stimulated microconidia germination of the tomato pathogens *F. oxysporum* f. sp. *lycopersici* and *F. oxysporum* f. sp. *radicis-lycopersici* and the level of stimulation was affected by plant age (Steinkellner et al., 2005). Moreover, root exudates can be detected by fungal pathogens enabling fungal hyphae to orient their growth towards the root. For example, the chemotropic response of *F. oxysporum* towards tomato roots was recently characterized and involves the catalytic activity of root‐secreted class III peroxidases (Turrà et al., 2015). Under favorable environmental conditions, soilborne pathogens invade plants through the root system and in most cases roots and other belowground parts are directly affected; however, symptoms are often visible on above ground parts of plants (Koike et al., 2003). Plants infected by soilborne pathogens suffer from root rots, inhibition of root development, stunted growth, seedling damping-off, stem and collar rots, wilting or even plant death (De Coninck et al., 2015; Katan, 2017). Diseases caused by soilborne plant pathogens are notoriously difficult to control for several reasons: many soilborne pathogens produce persistent resting structures that can survive in the soil for many years even in the absence of a susceptible host (Katan, 2017); measures targeting resting structures (e.g. chemical fumigation) are unsuitable for large-scale application due to public health and environmental issues and ban on chemical fumigants (Yadeta and Thomma, 2013); application of pesticides is often insufficient because of the poor accessibility in soil matrix (De Coninck et al., 2015); some of the soilborne pathogens infect a wide range of host plants rendering cultural control measures ineffective (Antoniou et al., 2017). Moreover, in order to establish a parasitic relationship with the plants, pathogens must interact with the complex rhizosphere community that also influences the outcome of the infection (Raaijmakers et al., 2009). Pathogens are negatively affected by co-inhabiting microorganisms through antibiosis and competition for nutrients, processes that usually involve secreted molecules. Snelders et al. (2018) proposed that pathogens can fight back by delivering effector proteins which target the rhizosphere communities instead of the plant to ultimately facilitate host colonization by the pathogen. Soilborne pathogens include species of fungi, oomycetes, bacteria, viruses and nematodes (Katan, 2017). The most important soilborne fungal pathogens are *Fusarium oxysporum* (Michielse and Rep, 2009), *Fusarium solani* (Coleman, 2016), *Rhizoctonia solani* (Gonzalez et al., 2011), *Verticillium* spp. (Klosterman et al., 2009), and *Sclerotinia sclerotiorum* (Bolton et al., 2006) and destructive soilborne oomycetes are *Phytophthora* spp. (Van West et al., 2003; Lamour et al., 2012) and *Pythium* spp. (Van West et al., 2003). Among many soil bacteria that are beneficial, there are only a few groups that infect the plant roots. Examples are *Ralstonia solanacearum* (Peeters et al., 2013) and the causal agent of crown gall *Agrobacterium tumefaciens* (Anand et al., 2008) that require a natural opening or wound to penetrate into the plant and cause infection. Only a small number of viruses can infect roots and like bacteria, they require an opening to achieve penetration. They generally survive only in the living tissues of the host plant or in their vectors. In soil, viruses are transmitted by zoosporic fungi (Campbell, 1996) or by nematodes (Brown et al., 1995).

### How Do Plants Select Microbes and Defend Against Pathogens

#### Effect of Root Exudates on Root-Associated Microbiome

Plants produce and exude *via* their roots various metabolites that can affect the assembly of the root microbiome before even microbes reach the root surface where they confront with the plant immune system (Sasse et al., 2017). The age and developmental stage of the plant influence exudation and subsequently the microbes proliferating around roots. Exudates of Arabidopsis plants collected at different plant age varied in sugar levels which affected accordingly microbial functions related with sugar and secondary metabolism (Chaparro et al., 2013). It was also shown that Arabidopsis plants during the early and late stage of their development can influence the abundance of Actinobacteria, Bacteroidetes and Cyanobacteria and microbial activity as well (Chaparro et al., 2014). Functions aligning with pathogens were more represented at early developmental stages while later developmental stages were dominated by functions related with antibiosis and chemotaxis and aligned to beneficial microbes, suggesting a selective pressure during plant aging towards microbes that provide their hosts with important services. In this direction, a recent study elegantly demonstrated that exudates change during the growth cycle of *Avena barbata* with sucrose levels are high at earlier stages while amino acids and defense molecules are released more at later developmental stages (Zhalnina et al., 2018). Using exometabolomics, this study showed that selected metabolites including aromatic organic acids (nicotinic, shikimic, salicylic, cinnamic, and IAA) are responsible for the proliferation or not of specific microbes around the roots during the different growth stages of the host plant (Zhalnina et al., 2018).

Different rhizodeposits have been shown to influence the microbiome composition. Studies on how plants select root-associated microbes/microbiota are summarized in **Table 1**. Biosynthesis of aliphatic and indolic glucosinolates, that are components of the chemical defense of plants, occurs in the vascular stele (Xu et al., 2017). Early studies demonstrated that root exudation of aliphatic glucosinolates can affect the rhizospheric microbial communities (Bressan et al., 2009), while indolic glucosinolates accumulate in Arabidopsis root upon pathogen infection (Bednarek et al., 2005). Combinations of exudates collected from Arabidopsis plants growing *in vitro* and applied in soil in the absence of plants revealed differential effects of phenolic compounds on the abundance of bacterial taxa (Badri et al., 2013). More specifically, phenolics seemed to have the biggest effect on the growth and attraction of bacterial operational taxonomic units (OTUs), followed by amino acids and sugars. A role of phenolics in affecting soil microbial diversity was also demonstrated with an Arabidopsis ABC transporter mutant (*abcg30*) which releases more phenolics but shows a reduced export of sugars (Badri et al., 2009). In soil in which *abcg30* plants were grown, an increased abundance of PGPR or bacteria involved in heavy metal remediation was observed compared to wild type Col-0 plants, suggesting a role for phenolics in attracting beneficial microbes. More recent studies suggested that coumarins, which are also phenolic compounds, can shape the rhizosphere microbiome and display differential toxicity against beneficial and pathogenic microbes (Stringlis et al., 2018b; Stringlis et al., 2019a; Voges et al., 2019). Next to phenolics, more chemical players have been found to contribute in the balance between roots and the microbiome, including benzoxazinoids (Hu et al., 2018; Cotton et al., 2019), triterpenes (Huang et al., 2019), and camalexin (Koprivova et al., 2019). Other naturally occurring exudates, like flavonoids and strigolactones, act as signaling compounds for the establishment of well-characterized symbiotic interactions of plant hosts with rhizobia and AMF (Akiyama et al., 2005; Subramanian et al., 2007). Moreover, border cells and border-like cells that are forming an extra root layer between the root tip and soil have been shown to affect a group of soilborne bacteria, because of proteins synthesized and released through them (Driouich et al., 2013). Arabinogalactan proteins were identified among the secreted molecules and were found to regulate *Rhizobium* and *Agrobacterium* attachment on roots (Gaspar et al., 2004; Vicre et al., 2005; Xie et al., 2012). Different parts of the root can release a different blend of exudates that can favor the colonization by selected members of the microbiome (Baetz and Martinoia, 2014). Studies using modern techniques like microfluidics and bacterial biosensors responsive to selected root exudates have revealed the preferential colonization of the root elongation zone and of lateral roots by bacteria of the genera *Bacillus* and *Rhizobium* (Massalha et al., 2017; Pini et al., 2017).

**Table 1:** Representative studies where plants under different stresses can select/modulate the assembly of the root-associated microbiome. For each study (when possible) the trigger leading to plant activity that modulates the microbiome, the identified mechanism of action, the effect on the microbiome, the host plant and the reference is mentioned.

Trigger	Mechanisms	Effect	Host	Reference
**Pathogen-triggered**				
*Fusarium oxysporum* f. sp. *lycopersici*	Disease -induced recruitment from suppressive compost	Enrichment of Proteobacteria, Actinobacteria, and Firmicutes (*Bacillus*)	Tomato	Antoniou et al., 2017
*Hyaloperonospora arabidopsidis/Pseudomonas syringae* pv. *tomato*	Legacy-mediated development of soil suppressiveness	Assemblage of beneficial rhizosphere microbiome	Arabidopsis/Tomato	Berendsen et al., 2018/ Yuan et al., 2018
*Rhizoctonia solani*	Activation of bacterial stress responses and activation of antagonistic traits that restrict pathogen infection	Shifts in microbiome composition and enrichment of *Oxalobacteraceae*, *Burkholderiaceae*, *Sphingobacteriaceae,* and *Sphingomonadaceae*	Sugar beet	Chapelle et al., 2016
*Botrytis cinerea*	Chemoattraction induced by root-exuded peroxidases and oxylipins	Attraction of *Trichoderma harzianum and* inhibition of *Fusarium oxysporum*	Tomato; Cucumber	Lombardi et al., 2018
*Rhizoctonia solani*	Pathogen-induced taxa enrichment from suppressive soils	Recruitment of specific taxa from rhizosphere of sugar beet infected with *Rhizoctonia solani*	Sugar beet	Mendes et al., 2011
*Pseudomonas syringae* pv. *tomato*	Root-secreted malic acid	Recruitment of *Bacillus subtilis* FB17	Arabidopsis	Rudrappa et al., 2008
*Fusarium oxysporum* f. sp. *lini*	Disease-induced recruitment of beneficial microbes from *Fusarium* suppressive soils	Increase of taxa associated to Fusarium wilt suppressiveness	Flax	Siegel-Hertz et al., 2018
Huanglongbing (HLB) caused by *Candidatus Liberibacter* spp.	Putative mechanisms: HLB significantly altered the structure or functional potential of the citrus endosphere	Decrease in abundance of taxa and loss of functions in the rhizoplane-rhizosphere enriched microbiome of HLB- infected citrus roots	Citrus	Zhang et al., 2017
**Insects-triggered**				
Aphids	Elicitation of plant immunity *via* SA/JA systemic signaling and expression of pathogenesis-related (PR) proteins in roots	Recruitment of the beneficial bacteria *Bacillus subtilis* and decrease of the population of *Ralstonia solanacearum*	Pepper	Lee et al., 2012
Whitefly	Whitefly infestation elicited SA and JA signaling in above and below ground tissues and overexpression of PR genes in the roots resulting in a differential microbiome assembly	The differential microbiome assembly induced resistance against to *Xanthomonas axonopodis* pv. *vesicatoria* and *Ralstonia solanacearum*	Pepper	Yang et al., 2011
**Abiotic stress/nutrient deficiency-triggered**				
Phosphate deficiency	Phosphate starvation response *via* PHR1 and PHL1 and PHO2	Differential assemblage of bacterial and fungal microbiota	Arabidopsis	Castrillo et al., 2017/ Fabianska et al., 2019
Gradients of phosphate, salinity, pH, temperature	-	Assembly of different modules of co-occurring strains	Arabidopsis	Finkel et al., 2019
wounding; salt stress	Chemoattraction induced by root-exuded peroxidases and oxylipins	Exudates attracted *Trichoderma harzianum* and showed deterrent activity against *Fusarium oxysporum*	Tomato; Cucumber	Lombardi et al., 2018
Iron deficiency/colonization by PGPR	Increased accumulation and secretion of the coumarin scopoletin exerts selective antimicrobial activity in rhizosphere	Differential microbiome assembly, repelling potential against phytopathogens and thus, recruiting potential beneficial microbes	Arabidopsis	Stringlis et al., 2018b
Iron deficiency	Catecholic coumarins show differential antimicrobial activity	Shift in microbial composition of SynCom *in vitro*	Arabidopsis	Voges et al., 2019
**Endogenous/exogenous plant-derived molecules-triggered**				
-	Overexpression of genes involved biosynthesis and transport of root-exuded secondary metabolites	Greater abundance of potentially beneficial bacteria	Arabidopsis	Badri et al., 2009
-	Differential exudation of root secondary metabolites regulated by Benzoxazinoids (BXs)	Enrichment of *Methylophilaceae, Nitrosomonadaceae, Oxalobactereraceae, Syntrophobacteriaceae,* and *Gaiellaceae*	Maize	Cotton et al., 2019
-	Benzoxazinoids (BXs) drive plant-soil feedback	BXs shape the microbiota of the next generation of plants	Maize	Hu et al., 2018
-	Differential secretion of triterpene-derived metabolites by altering triterpene gene cluster	Differential assembly of Arabidopsis root microbiome	Arabidopsis	Huang et al., 2019
-	Microbial sulfatase cleaves root-exuded sulfate esters produced by the camalexin biosynthetic pathway	Stimulation of microbial sulfatase activity in soil and is required for the plant growth-promoting effects of several bacterial strains	Arabidopsis	Koprivova et al., 2019
-	Assembly of differential microbiome between tomato cultivars susceptible and resistant to *Ralstonia solanacearum*	Enrichment of *Flavobacterium* in the microbiome of tomato cultivars resistant to *Ralstonia*, *Flavobacterium* application confers resistance to susceptible cultivar	Tomato	Kwak et al., 2018
SA	Compromised innate immune system impairing SA biosynthetic pathway	SA-dependent modulation of root microbiome and enrichment of *Flavobacterium*, *Terracoccus,* and *Streptomyces* in SA-treated roots and bulk soils	Arabidopsis	Lebeis et al., 2015
-	DIMBOA Benzoxazinoids (BXs) induce chemotaxis-associated genes in *Pseudomonas putida*	Enhanced rhizosphere colonization by *P. putida*	Maize	Neal et al., 2012
ACC; JA	ACC and JA application, induced altered expression of PRR and RLK and cell wall biosynthesis and maintenance related genes	Inhibition of the secondary stage of root colonization by *Laccaria bicolor*	Poplar	Plett et al., 2014b

#### Structural Root Defenses and Microbiome

Plants have developed various ways to restrict microbial growth and colonization on plant tissues, once microbes overcome niche competition with other microbes in the rhizosphere and can successfully grow in root exudates. In leaves, an armory of structural and chemical defense mechanisms have evolved to prevent disease caused by colonization of harmful microbes inside plant tissues (Senthil-Kumar and Mysore, 2013). These structural defense components include the cuticle, lignin, suberin and deposition of callose and are also present in the roots. Roots are plant organs characterized by radial organization where each concentric layer corresponds to a different tissue (Wachsman et al., 2015). Lignin fortifies the xylem of *Arabidopsis* roots (Van De Mortel et al., 2008; Naseer et al., 2012) and going outwards from the root core, lignin-composed Casparian strips (CS) and the hydrophobic polymer suberin make the endodermis a barrier between the xylem and the soil (Naseer et al., 2012; Geldner, 2013). Recognition of microbes or of microbial elicitors can induce callose deposition in the epidermal cells of the root (Millet et al., 2010; Jacobs et al., 2011; Hiruma et al., 2016). Finally, cutin as a waxy polymer of the cuticle coating the epidermis, has barrier-like properties like suberin and is present in the primary and lateral roots (Berhin et al., 2019). Evidence suggests that plant defense components exert some selective pressure on the microbes that can colonize the inner tissues of the root. The first seminal studies on the root microbiome field demonstrated that the endosphere microbiota is a fraction of the rhizosphere microbiota, and structural defense components might have a role in this observation (Bulgarelli et al., 2012; Lundberg et al., 2012). Other structural modifications of the root system like emergence of lateral roots or formation of root hairs might be involved in creating micro-niches that host distinct subsets of the root microbiota. A study in barley comparing wild type and mutant plants for root hair formation revealed that the microbial community in root hair mutants was simpler and less diverse compared to the microbial communities assembled in the roots of wild type barley plants (Robertson-Albertyn et al., 2017). Despite the presence of structural defense components in roots and their dynamic contribution in plant growth, information on their role in the assembly of the root microbiome is still limited.

#### Interplay Between Plant Immunity and the Microbiome

##### Root Immune System

As already mentioned in this review, soil microbial populations consist of a mix of beneficial and pathogenic microbes. Hence, plants need to successfully recognize them and subsequently reprogram their defense strategies to allow or block their colonization (Zamioudis and Pieterse, 2012; Yu et al., 2019a). To effectively and timely perceive microbial signals, plants have evolved a multilayered detection system that leads, depending on the trigger, to the activation of downstream defense responses (Dodds and Rathjen, 2010). In the first layer of this defense system, surface-localized pattern recognition receptors (PRRs) perceive conserved microbe-derived molecules, called microbe-associated molecular patterns (MAMPs). In Arabidopsis, some MAMP/PRR pairs are well defined (Couto and Zipfel, 2016). Bacterial flagellin and the immunogenic epitope of flagellin flg22 are perceived by receptor kinase FLAGELLIN-SENSING 2 (FLS2) (Gomez-Gomez and Boller, 2000), while ELONGATION FACTOR-TU RECEPTOR (EFR) recognizes bacterial elongation factor Tu and its derived immunogenic peptide elf18 (Kunze et al., 2004). Additionally, CHITIN ELICITOR RECEPTOR KINASE 1 (CERK1) and LYSIN MOTIF CONTAINING RECEPTOR-LIKE KINASE 5 (LYK5) recognize hepta- or octamers of the fungal elicitor chitin (Miya et al., 2007; Cao et al., 2014). The recognition of a MAMP leads to the induction of immune responses in the host plant that constitute the first layer of defense referred to as MAMP-triggered immunity (MTI). Based on their timing, the activated immune responses range from instant [medium alkalization, oxidative burst (ROS), protein phosphorylation] and early (ethylene biosynthesis, defense gene activation) to late (callose deposition and growth inhibition) (Boller and Felix, 2009). All these processes aim to halt any further growth of a microbe on/in plant tissues and have been elucidated by the extensive study of pathogen perception in the aerial plant tissues. During the last decade, many studies have shown that roots can perceive MAMPs and generate MAMP-specific responses such as callose deposition, camalexin biosynthesis, and induction of defence‐related genes similar to leaves (Millet et al., 2010; Jacobs et al., 2011; Wyrsch et al., 2015; Poncini et al., 2017; Stringlis et al., 2018a; Marhavy et al., 2019). Constitutive activation of PRRs in microbe- and elicitor-enriched environments like roots and the surrounding rhizosphere could result in unnecessary MTI that in turn could cause growth and yield inhibition of plants (Gomez-Gomez et al., 1999; Vos et al., 2013). For this, different researchers aimed to define the involvement of different plant organs in flg22 perception by its receptor FLS2 (Beck et al., 2014) and the contribution of different root tissues in the induction of MTI upon flg22 elicitation (Wyrsch et al., 2015). Interestingly, inner tissues show higher expression of the FLS2 receptor and stronger MAMP responses (ROS production and induction of defense genes) compared to epidermal tissues. However, it's not only the plant side that adapts to the presence of MAMPs, but the microbes themselves adapt to the presence of PRRs. Only a small fraction of the genomes of the culturable microbiome of Arabidopsis (3%–6%) contains genes coding for flg22 or elf18 peptides, while the peptide cold shock protein 22 (csp22) recognized by Solanaceae and not by Arabidopsis is present in 25% of the isolated Arabidopsis-associated microbes (Wang et al., 2016; Hacquard et al., 2017). This suggests that the presence of PRRs in roots exerts a selective pressure on the root-associated microbes that need to develop mechanisms to mask the presence of their MAMPs and achieve colonization. Some PRRs can also identify “self” molecules known as host-derived damage-associated molecular patterns (DAMPs). In response to cellular rupture by nematodes or fungal attack, DAMPs are released and can induce strong tissue specific responses in the roots of Arabidopsis (Poncini et al., 2017; Marhavy et al., 2019). Considering the potential of DAMPs to induce stronger defense responses in the roots compared to MAMPs (Poncini et al., 2017), their role in the assembly of the root microbiome and on how plants discriminate between beneficial and pathogenic root colonizers should be expected.

###### Suppression of Root Defenses by Beneficial Microbes

Signaling pathways of defense hormones SA and JA have been long-involved in responses of plants to infection by pathogens or colonization by beneficial microbes (Pieterse et al., 2012; Zamioudis and Pieterse, 2012; Pieterse et al., 2014) and studies using mutants for these hormonal pathways have demonstrated their role in shaping the root microbiome (Carvalhais et al., 2015; Lebeis et al., 2015). Beneficial members of the root microbiota have developed different strategies to suppress MTI and/or manipulate the homeostasis of defense hormones to achieve colonization and provide their host with benefits (Zamioudis and Pieterse, 2012; Yu et al., 2019a). Symbiotic mycorrhizal and ectomycorrhizal fungi *Rhizophagus irregularis* and *Laccaria bicolor* secrete mutualism effectors that manipulate ET and JA hormonal signaling pathways (Kloppholz et al., 2011; Plett et al., 2011; Plett et al., 2014a; Plett et al., 2014b), while effectors of endophytic fungus *Serendipita indica* target JA signaling to achieve defense suppression (Jacobs et al., 2011; Akum et al., 2015). JA signaling is also upregulated by PGPF *Trichoderma* spp. to suppress activation of immune responses during early colonization of the root (Brotman et al., 2013). Beneficial bacteria employ different strategies to manipulate the host and accomplish colonization. The type III secretion system (T3SS) is important in the establishment of symbiosis between rhizobia and their legume partners (Zamioudis and Pieterse, 2012). T3SS is a multicomponent apparatus that Gram negative bacteria, mostly pathogenic, use to secrete effector molecules into host cells aiming to restrict the defense responses mounted due to their recognition and achieve host colonization (Galan and Collmer, 1999). *Sinorhizobium fredii* HH103 with defective T3SS is unable to suppress SA-dependent defenses and subsequently fails to promote nodulation on its legume host (Jimenez-Guerrero et al., 2015). Non-symbiotic PGPR such as *Pseudomonas fluorescens* SBW25, *Pseudomonas brassicacearum* Q8r1-96 and *Pseudomonas simiae* WCS417 and other root-associated Pseudomonads, are also equipped with T3SS, however its role in root colonization remains elusive (Preston et al., 2001; Mavrodi et al., 2011; Loper et al., 2012; Berendsen et al., 2015; Stringlis et al., 2019b). Nevertheless, beneficial microbes can employ other mechanisms independent of secretion systems to mask their presence in the rhizosphere. Pathogenic bacteria *Pseudomonas aeruginosa* and *Pseudomonas syringae* release the extracellular alkaline protease AprA which degrades flagellin monomers, and allows microbes to have their MAMPs undetected by the immune system of both mammals and plants (Bardoel et al., 2011; Pel et al., 2014). Plant-beneficial bacteria have AprA homologs in their genomes so a role of this protease in their interaction with roots is possible (Pel et al., 2014). More recently, Yu et al. (2019b) suggested another mode of plant manipulation where beneficial rhizobacteria of the genus *Pseudomonas* spp. produce organic acids during root colonization that lower the environmental pH and in turn suppress root immune responses following recognition of the flg22 peptide.

### Phenomena Where Selection Occurs

#### Building Up of Disease Suppressiveness

Soil microbial communities provide silently their valuable services in terrestrial ecosystems by increasing ecosystem resilience, making soil more resistant to any disturbance-induced damages due to environmental changes (Berendsen et al., 2012). Disease suppression is a well-known microbiome-mediated phenomenon that provides a first line of defense against infections by the soilborne pathogens (Weller et al., 2002). Disease suppressive soils have been originally defined as “soils in which the pathogen does not establish or persist, establishes but causes little or no damage, or establishes and causes disease for a while but thereafter the disease is less important, although the pathogen may persist in the soils” (Baker and Cook, 1974). In contrast, in conducive soils the disease occurs readily. Two types of soil suppressiveness have been characterized: “general” and “specific” suppression. In general suppression, growth and activity of pathogens are inhibited to some extent and the suppressiveness is attributed to the antagonistic activity of the collective microbial community that is often associated with competition for available resources (Mazzola, 2002; Weller et al., 2002; Cook, 2014). General suppressiveness is enhanced by the incorporation of organic amendments or other management practices that increase the total microbial activity and competition in the soil (Weller et al., 2002; Bonanomi et al., 2010). It is often effective against a broad range of pathogens and is not transferable between soils (Cook and Rovira, 1976; Weller et al., 2002). General suppressiveness is a pre-existing characteristic of soils and is fundamentally microbiological in nature (Weller et al., 2002; Raaijmakers and Mazzola, 2016). Specific suppression occurs when individual species or specific subsets of soil microorganisms interfere with the infection cycle of a pathogen (Weller et al., 2002; Berendsen et al., 2012). The biotic nature of specific suppression is also demonstrated as it can be eliminated through soil pasteurization or biocides. In contrast to general suppressiveness, specific suppressiveness can be transferred by introducing very small amounts (1%–10%) of suppressive soil into a conducive soil (Cook and Rovira, 1976; Mendes et al., 2011; Raaijmakers and Mazzola, 2016; Schlatter et al., 2017). Specific suppression is superimposed over the general suppression and is more effective (Berendsen et al., 2012). In some soils specific suppression is retained for prolonged periods even when soils are left bare, whereas in other soils it is induced by continuous monoculture of a susceptible host after a disease outbreak (Berendsen et al., 2012; Raaijmakers and Mazzola, 2016). Induction of specific suppression requires multilateral interactions between plants, soil microbiome and pathogens and is mechanistically complex. The interaction between plant and pathogen that occurs before a disease outbreak may induce the release of pathogen- or plant-derived metabolites that lead to alterations in microbiota composition and activation of pathogen-suppressive microorganisms (Chapelle et al., 2016). In recent years, many studies using new culture-independent technologies started to unravel the identity of responsible microorganisms in disease suppressive soils (Gomez Exposito et al., 2017). For instance, suppressiveness towards *Verticillium dahliae* was mainly associated with higher abundances of Actinobacteria and *Oxalobacteraceae* (Cretoiu et al., 2013). Another study regarding fungi revealed significant differences in the fungal community composition between suppressive and non-suppressive soil for the disease caused by *R. solani* AG 8; *Xylaria*, *Bionectria,* and *Eutypa* were more abundant in the suppressive soil whereas *Alternaria* and *Davidiella* dominated the non-suppressive soil (Penton et al., 2014). Also, higher abundances of the Phyla Actinobacteria, Proteobacteria, Acidobacteria, Gemmatimonadetes, and Nitrospirae were found in soil with specific suppressiveness to Fusarium wilt of strawberry (Cha et al., 2016). More recently, it was shown that fungal and bacterial diversity differed significantly between a suppressive and a conducive soil of Fusarium wilt whereas several of the fungal and bacterial genera known for their activity against *F. oxysporum* were detected exclusively or more abundantly in the Fusarium wilt-suppressive soil (Siegel-Hertz et al., 2018). Interestingly, studies analyzing the rhizobacterial community composition in soils suppressive or conducive to *R. solani* revealed that relative abundance of specific bacterial taxa is a more important indicator of suppressiveness than the exclusive presence or absence of specific bacterial families (Mendes et al., 2011; Chapelle et al., 2016). In a study by Hu et al. (2016) defined *Pseudomonas* species consortia were introduced into naturally complex microbial communities to assess the importance of the *Pseudomonas* community diversity for the suppression of *R. solanacearum* in the tomato rhizosphere. Only the most dense and diverse *Pseudomonas* communities reduced pathogen density in the rhizosphere and decreased the disease incidence due to both intensified resource competition and interference with the pathogen. Recently, Wei et al. (2019) demonstrated that the composition and functioning of the initial soil microbiome predetermines future disease outcome of *R. solanacearum* on tomato plants. Plant survival was associated with specific bacterial species, including the highly antagonistic *Pseudomonas* and *Bacillus* bacteria together with specific rare taxa. The mechanism behind the suppression could be the production of antibiotics, as high abundance of genes encoding non-ribosomal peptide and polyketide synthases was found in the initial microbiomes associated with healthy plants. Intriguingly, they also demonstrated that this capacity can be transferred to the next generation of plants through soil transplantation opening a new avenue of exploiting microbiomes for disease resistance.

#### Microbiome Modulation by Coumarins, Benzoxazinoids, and Other Root-Exuded Molecules

##### Coumarins

Coumarins are phenolic compounds produced *via* the phenylpropanoid pathway and have been extensively studied for their role in disease resistance (Stringlis et al., 2019a) but also for their involvement in responses of dicotyledonous plants to iron deficiency (Tsai and Schmidt, 2017a). Coumarins are produced when iron is unavailable in the soil around the roots and their exudation increases to make iron more available before it is imported inside the roots (Tsai and Schmidt, 2017b; Tsai and Schmidt, 2017a). Coumarins with pronounced production/exudation in response to iron deficiency are scopolin, scopoletin, esculin, esculetin, fraxetin and sideretin (Jin et al., 2007; Rodriguez-Celma et al., 2013; Fourcroy et al., 2014; Schmid et al., 2014; Schmidt et al., 2014; Fourcroy et al., 2016; Rajniak et al., 2018; Tsai et al., 2018). Recent studies have suggested their role also in shaping microbiome composition around the roots (Stringlis et al., 2018b; Voges et al., 2019). Stringlis et al. (2018b) showed that both under iron deficiency and colonization of roots by beneficial rhizobacteria that induce ISR, there is increased accumulation of coumarins inside the roots. Components of the production and exudation of coumarins in this study were genes with a key role in ISR, such as the root-specific transcription factor MYB72 and beta-glucosidase gene *BGLU42* (Verhagen et al., 2004; Van Der Ent et al., 2008; Zamioudis et al., 2014; Stringlis et al., 2018b). More specifically, in *myb72* mutant plants no coumarin accumulation was observed inside the roots, while in *bglu42* mutant plants there was reduced exudation of coumarin scopoletin. Analysis of the rhizosphere microbiomes in these mutants plants, the coumarin biosynthesis mutant *f6'h1* (Kai et al., 2008; Schmid et al., 2014) and wild-type plants revealed that coumarins can affect the composition of the microbiome around the roots (Stringlis et al., 2018b). There was increase in the relative abundance of Proteobacteria but decrease of Firmicutes in the *f6'h1* rhizosphere compared to wild-type plants rhizosphere. Further experiments showed that coumarin scopoletin was inhibiting the growth of soilborne pathogens whereas rhizobacteria that induce ISR were insensitive to its antimicrobial activity (Stringlis et al., 2018b; Stringlis et al., 2019a). Voges et al. (2019) showed that coumarins can shape the composition of a synthetic bacterial community inoculated in *in vitro* grown plants and there was enrichment of a *Pseudomonas* strain in *f6'h1* compared to wild-types plants growing under iron deficiency. In this study, it was suggested that the antimicrobial effect of catecholic coumarins fraxetin and sideretin, produced downstream of scopoletin (Rajniak et al., 2018; Tsai et al., 2018), are due to the hydrogen peroxide deriving from catecholic coumarins at conditions of iron deficiency (Voges et al., 2019).

##### Benzoxazinoids

Benzoxazinoids are a class of compounds, quite abundant in the roots of maize, with a documented role in the attraction of beneficial microbes in the rhizosphere (Neal et al., 2012) and the defense responses of plants to various pathogenic threats (Ahmad et al., 2011). Recently, studies have focused on characterizing how benzoxazinoids can shape the assembly of root-associated bacterial and fungal communities (Hu et al., 2018; Cotton et al., 2019). Hu et al. (2018) using a benzoxazinoids deficient maize mutant observed that different bacterial and fungal communities assemble in the roots of the mutants compared to wild-type maize. Despite the prominent changes in bacterial and fungal microbiome the authors didn't assess the effects of benzoxazinoids on specific bacterial/fungal taxa. Release of benzoxazinoids and the subsequent microbiome changes were sufficient to provide plants of a next generation growing in this soil with protection against a herbivore insect. Next–generation maize plants growing in soil with and without benzoxazinoids displayed distinct bacterial and fungal communities both in the root and the rhizosphere. Actinobacteria OTUs and some Ascomycota and Glomeromycota OTUs were mostly responsible for root and rhizosphere separation but the effects on plant fitness were more strongly associated with changes in bacteria than fungi in the rhizosphere of these next-generation plants (Hu et al., 2018). There was increase of a subset of Proteobacteria in soils with benzoxazinoids, while Chloroflexi OTUs were enriched in soils without benzoxazinoids. In the case of fungal communities, Ascomycota OTUs were present in both soils with and without benzoxazinoids. Interestingly, Glomeromycota OTUs seemed to be less abundant in soils with benzoxazinoids. In the study by Cotton et al. (2019), the effect of benzoxazinoids on the metabolomic profile of roots and microbiome assembly was assessed. Metabolomic profiles of mutants in benzoxazinoids production were different compared to those of wild type plants, indicating a role of benzoxazinoids in the metabolic response of maize roots. The microbiome analysis revealed enrichment or depletion of bacterial and fungal OTUs between the rhizospheres of wild type and mutant plants and the authors correlated the changes in the microbial abundance with metabolites present in the roots of wild type and mutant plants (Cotton et al., 2019). Studies like those presented herein on coumarins and benzoxazinoids enrich our understanding on how specific exudates shape root-associated microbial communities, and unlocking how a beneficial microbiome can be selected *via* exudation could allow us to breed for plants that can manipulate their microbiome to maximize growth and health benefits (Vannier et al., 2019).

##### Triterpenes and Camalexin

As already mentioned in section *Effect of Root Exudates on Root-Associated Microbiome*, triterpenes and camalexin were recently found to be involved in microbiome shaping (Huang et al., 2019; Koprivova et al., 2019). Triterpenes are products of plant metabolism with involvement in disease resistance and with antimicrobial activity (Papadopoulou et al., 1999). Triterpenes are synthesized *via* the mevalonate pathway and can accumulate in plant tissues as triterpene glycosides (Thimmappa et al., 2014). Huang et al. (2019) observed that triterpenes thalianin and arabidin are produced in roots and biosynthetic genes for their production are induced following treatment of roots with MeJA. Microbiome analysis of thalianin and arabidin mutants and wild-type plants revealed the assembly of distinct root microbial communities in the absence of triterpenes. These differences were explained by the enrichment of Bacteroidetes and the depletion of Deltaproteobacteria in the roots of triterpene mutants compared with the roots of wild type plants (Huang et al., 2019). In the study of Koprivova et al. (2019), the authors performed a genome wide association study (GWAS) and measured microbial sulfatase activity in the soil where 172 accessions of Arabidopsis were grown. Through this screen the authors found single-nucleotide polymorphisms (SNPs) explaining differences in microbial sulfatase activity. Some of these SNPs were in gene *CYP71A27* and a mutant of this gene displayed reduced microbial sulfatase activity and impaired production of antimicrobial compound camalexin. Interestingly, the authors observed that beneficial rhizobacteria could promote growth in wild-type plants but only beneficial rhizobacteria without sulfatase activity could promote growth in *cyp71a27* mutants. The fact that beneficial rhizobacterium *Pseudomonas* sp. CH267 could promote growth in wild-type plants but not in nine Arabidopsis accessions with variation in the amino acid sequence of CYP71A27, suggested that camalexin is required in the interaction of roots with microbes in order the plants to have a benefit (Koprivova et al., 2019).

#### “Cry for Help” During Infection of Plants

Plants experiencing infection by phytopathogens or insects, actively recruit beneficial members from the rhizosphere microbiota that will help them overcome biotic stresses, a phenomenon defined as “cry for help” (Bakker et al., 2018). Studies have shown that the build-up of a beneficial microbial community in the root is mediated by changes in gene expression and alterations in root exudation responsive to pathogen attack (**Figure 1**). Rudrappa et al. (2008) showed that infection of Arabidopsis leaves by *Pseudomonas syringae* pv. *tomato* (Pst) induced the root exudation of malic acid that in turn favored the recruitment of the beneficial *Bacillus subtilis* strain FB17 which triggers ISR in Arabidopsis against Pst. Tomato plants experiencing different stresses produced exudates that acted as chemoattractants for the beneficial fungus *Trichoderma harzianum* (Lombardi et al., 2018). Other studies have shown that aphid feeding or whitefly infestation of pepper and tobacco leaves can cause a transcriptional reprogramming in roots and changes in the root microbiome composition which makes plants more resistant to foliar and soilborne pathogens (Yang et al., 2011; Lee et al., 2012; Lee et al., 2018). Recently, Berendsen et al. (2018) demonstrated that Arabidopsis leaf infection by the biotrophic oomycete *Hyaloperonospora arabidopsidis* (Hpa) can lead to the enrichment of three bacterial taxa (*Xanthomonas* spp., *Stenotrophomonas* spp., and *Microbacterium* spp.) in the rhizosphere. Isolation of these microbes and inoculation of Arabidopsis showed that these three microbes together could induce ISR against Hpa and promote plant growth, indicating the active recruitment of beneficial microbes by infected plants. Microbiome changes were also apparent in Arabidopsis infected with *Pseudomonas syringae* and those changes were attributed to changes in root exudation (Yuan et al., 2018). In these studies, the beneficial effect in plant health due to microbiome changes could be transferred to the offspring of the infected plants that displayed increased levels of resistance to these pathogens (Berendsen et al., 2018; Yuan et al., 2018). These findings indicate that in soils with infected plants changes in exudation and the microbiome lead to the build-up of a microbial legacy that is inherited to the next generations of plants growing in this soil and favors their survival under phytopathogenic pressure (Bakker et al., 2018). Considering the continuity of plant-pathogens interactions during the lifetime of a plant in a field, a functional “loop” should be in action: when plants experience stress they respond with changes in exudation that can favor the selection of beneficial microbial members from the rhizosphere which in turn can help the plants deal with the stress (Liu et al., 2019a). Future studies should elucidate how different exudates contribute in the microbial recruitment and the subsequent soilborne legacy described above, considering the involvement of coumarins (Stringlis et al., 2018b; Stringlis et al., 2019a), malic acid (Rudrappa et al., 2008), benzoxazinoids (Hu et al., 2018; Cotton et al., 2019), and camalexin (Koprivova et al., 2019) in the selection of beneficial microbes in the rhizosphere.

#### Rhizosphere Microbiome as a Source of Benefits for the Plant

##### Beneficial Effects Against Biotic Stresses

It is well documented that plant genotype exerts strong influence on the overall composition of root associated communities through plant root exudates (Bulgarelli et al., 2012; Badri et al., 2013; Matthews et al., 2019). Recent evidence suggest that root exudates attract beneficial and pathogen-suppressing microbes or reshape microbiome assembly in the plant rhizosphere to suppress disease symptoms (Kwak et al., 2018; Mendes et al., 2018). The study of Mendes et al. (2018) using common bean cultivars with variable levels of resistance has shown that rhizobacteria belonging to *Pseudomonadaceae*, *Bacillaceae*, *Solibacteraceae,* and *Cytophagaceae* families were more abundant in the rhizosphere of the Fusarium-resistant cultivar. Kwak et al. (2018) analyzed the rhizosphere microbiomes of a resistant and a susceptible tomato variety to the soilborne pathogen *R. solanacearum* to assess the role of plant-associated microorganisms in disease resistance and proved that transplantation of rhizosphere microbiota from resistant plants suppressed disease symptoms in susceptible plants. By comparing the metagenomes of the rhizosphere from resistant and susceptible plants a flavobacterial genome was identified to be far more abundant in the resistant plant rhizosphere. The isolated flavobacterium could suppress *R. solanacearum* in pot experiments with a susceptible tomato variety suggesting that selection of native microbiota can protect plants from root pathogens. Recently, it was shown that in natural populations of Arabidopsis, the plants are protected against root-inhabiting filamentous eukaryotes because of the presence of the co-residing bacterial root microbiota that is essential for plant survival (Duran et al., 2018). In another microbiome study, the occurrence of potato common scab caused by *Streptomyces* was correlated with the composition and putative function of the soil microbiome (Shi et al., 2019). The community composition of the geocaulosphere soil samples revealed that *Geobacillus*, *Curtobacterium*, and unclassified *Geodermatophilaceae* were the most abundant genera that were significantly negatively correlated with the scab severity level, the estimated absolute abundance of pathogenic *Streptomyces*, and *txtAB* gene copy number (biosynthetic gene of the scab phytotoxin). In contrast, *Variovorax*, *Stenotrophomonas*, and *Agrobacterium* were the most abundant genera that were positively correlated with these three parameters.

Direct pathogen suppression by rhizospheric microorganisms has been extensively reported (Mendes et al., 2011; Santhanam et al., 2015; Cha et al., 2016; Hu et al., 2016). Pathogen growth is affected by several and highly diverse mechanisms including microbial competition (for resources or space) (Zelezniak et al., 2015), secretion of antimicrobial compounds (Chen et al., 2018; Helfrich et al., 2018; Stringlis et al., 2018b; Koprivova et al., 2019) and hyperparasitism (Parratt and Laine, 2018). As mentioned previously, members of the rhizosphere microbiome can alter plant growth by producing phytohormones which modulate endogenous plant hormone levels (Stringlis et al., 2018c). In a recent study, two synthetic microbial communities were designed and consisted of bacterial strains that show ACC deaminase activity and produce an array of hormones and enzymes *in vitro* and also show antimicrobial activity against *F. oxysporum* f. sp. *lycopersici*. Inoculation of these synthetic communities in a poor substrate enhanced the growth of tomato plants and reduced symptoms caused by *F. oxysporum* f. sp. *lycopersici* (Tsolakidou et al., 2019a). In another study, endophytic *Enterobacteriaceae* strains engineered to express ACC deaminase activity on the bacterial cell walls did not show any activity against a pathogenic strain of *Fusarium oxysporum* f. sp. *cubense in vitro.* However, they promoted banana plant growth and increased resistance to banana Fusarium wilt suggesting that engineering the interactions between plants with their microbiome can provide valuable tools to deal with plant pathogens that are difficult to control (Liu et al., 2019b). Pathogenic microbes can employ similar strategies with beneficial microbes to colonize their hosts. For example, overexpression of *ACC deaminase* gene in *V. dahliae* significantly lowered ACC levels in the roots of infected tomato plants and increased both its virulence and the fungal biomass in the vascular tissues of plants (Tsolakidou et al., 2019b). Therefore, future studies need to address how functions shared by both beneficial and pathogenic microbes are perceived by the plants and how plants can maintain a balance in the rhizosphere.

##### Beneficial Effects Against Abiotic Stresses

Accumulating evidence suggests that the rhizosphere microbiome is not only involved in coping with biotic stresses but is also involved in protection of plants against abiotic stresses (**Figure 1**). Rhizosphere bacteria have been shown to elicit so-called induced systemic tolerance to high salinity, drought and nutrient deficiency or excess (Yang et al., 2009; Rolli et al., 2015). A recent study found a diverse range of root-associated bacteria of soybean and wheat, including *Pseudomonas* spp., *Pantoea* spp., and *Paraburkholderia* spp., showing mechanisms involved in improved nutrient uptake, growth, and stress tolerance like phosphate solubilization, nitrogen fixation, indole acetic acid and ACC deaminase production (Rascovan et al., 2016). Accumulation of heavy metals, hydrocarbons and pesticides in soil can cause deterioration of soil properties and have negative impact on plant growth or make the plant unsuitable for consumption (Kuiper et al., 2004). Interestingly, Sessitsch et al. (2012) found enrichment of microbial functions for the degradation of aromatic compounds in the metagenomes of endophytes, highlighting a potential for bioremediation. Understanding how microbiome dynamics and functions can change in response to perturbations can open new avenues to engineer microbial communities also for bioremediation purposes (Perez-Garcia et al., 2016; Eng and Borenstein, 2019). Indeed, soil tillage and compost amendment of contaminated soils could stimulate the indigenous microbial communities which are naturally adapted to the pollutants of these soils (Ventorino et al., 2019). In another study the modification of the microbiota assemblage following the introduction of a natural and diverse microbiome transplant in an oil-contaminated soil led to more efficient contaminant degradation compared to the introduction of an artificial microbial selection (Bell et al., 2016). Phytoremediation is the use of plants to extract, sequester, or detoxify pollutants. This practice is often associated with the microbial bioremediation since the presence of plants can stimulate the microbial population in the rhizosphere, improve physical and chemical properties of the soil and increase contacts between microbes and soil contaminants (Kuiper et al., 2004). In a recent work, Fan and colleagues found that inoculation of *Robinia pseudoacacia* with rhizobia, significantly affected rhizosphere microbial population and functions and also improved the phytoremediation capacity of the plants (Fan et al., 2018).

##### Plant Microbiome as a Source of Variability in Plant Breeding

The efforts of plant breeding practices have always been directed towards the selection of desirable phenotypic traits, such as higher yield associated with improved edible characteristics. This domestication process, progressively led to the loss of allelic diversity, also named as genetic erosion of domesticated plants (Perez-Jaramillo et al., 2016; Pieterse et al., 2016). Recent studies indicated that in several plant species the rhizosphere microbiome composition may have been affected in domesticated plants as compared to their wild relatives (Perez-Jaramillo et al., 2017; Perez-Jaramillo et al., 2018; Pérez-Jaramillo et al., 2019). For common bean, it was shown that relative abundance of Bacteroidetes was increased in wild accessions whereas Actinobacteria and Proteobacteria were enriched in modern accessions and this shifting was associated with plant genotypic and specific root morphological traits (Perez-Jaramillo et al., 2017). Interestingly, the transition of common bean from a native to an agricultural soil led to a gain of rhizobacterial diversity and to a stronger effect of the bean genotype on rhizobacterial assembly (Pérez-Jaramillo et al., 2019). In a study using 33 strains of sunflower (*Helianthus annuus*) with varying degrees of domestication it was found that rhizosphere fungal communities were more strongly influenced by host genetic factors and plant breeding than bacterial communities. They also found that there was a minimal vertical transmission of fungi from seeds to adult plants (Leff et al., 2017). A survey of the bacterial community structure of 3 barley accessions also pointed to a small but significant role of the host genotype on root-associated community composition (Bulgarelli et al., 2015). Perez-Jaramillo et al. (2018) conducted a meta-analysis integrating metagenomics data of 6 independent studies with the aim of addressing whether plant domestication affected the composition of the root-associated microbiome in various crop plant species and observed consistent enrichment of Actinobacteria and Proteobacteria in modern varieties in contrast to the enrichment of Bacteroidetes in their wild relatives. This evidence indicates that modern agriculture may not utilize the full potential the associated microbiome may offer. In this framework, wild relatives have been suggested to provide new perspective into plant genes associated with microbiome assembly, and this knowledge could open new horizons for future breeding strategies (Perez-Jaramillo et al., 2018).

### Engineering Microbial Inoculants to Suppress Disease and Support Plant Growth: From the Lab to the Field

#### The Prospect of Using Synthetic Communities to Promote Plant Health

The successful application of microbial consortia as inoculants to protect plants from stresses and enhance their productivity relies mainly on the ability of microorganisms that show promise in the lab to overcome hurdles and retain their characteristics when applied in the field (Sessitsch et al., 2019). The rationale behind this strategy is twofold: the selection and combination i) of distantly related microorganisms with different or complementing characteristics tailored to promote plant growth and suppress pathogens, or tolerate different plant genotypes or environmental conditions (Compant et al., 2019), or ii) of closely related strains in order to expand the diversity of resources that these strains use (Wei et al., 2015; Hu et al., 2016). Species-rich communities are often more efficient and more productive than species-poor communities as they use limiting resources more efficiently (Loreau et al., 2001). For instance, the introduction of high diversity *Pseudomonas* consortia reduced *R. solanacearum* density in the rhizosphere of tomato plants and decreased the disease incidence due to interference and intensified resource competition with the pathogen. Interestingly, increasing diversity of the introduced *Pseudomonas* consortia also increased their survival (Hu et al., 2016). Furthermore, increasing the richness of *Pseudomonas* consortia resulted in enhanced accumulation of plant biomass and more efficient assimilation of nutrients in tomato plants; diversity effects were more important than the identity of the *Pseudomonas* strain and the observed plant growth promotion was associated with elevated production of plant hormones, siderophores, and solubilization of phosphorus *in vitro* (Hu et al., 2017). In contrast, increasing genotypic richness of *P. fluorescens* communities increased disproportionally the antagonistic interactions, causing community collapse and resulted in loss of *Medicago sativa* protection against the oomycete *Pythium ultimum* (Becker et al., 2012). It was recently proposed that microbial synthetic communities can be used as inoculants to produce plant growth substrates with desired characteristics such as biocontrol of targeted pathogens and plant growth promotion (Tsolakidou et al., 2019a). The composition of the synthetic communities was a determinant factor for the growth of plants and pathogen inhibition. The synthetic community consisting of different bacterial genera promoted the growth of tomato plants but failed to protect plants against Fusarium wilt. The synthetic community consisting of *Bacillus* isolates suppressed Fusarium wilt symptoms and enhanced tomato growth but to a lesser extent as compared to the more diverse synthetic community (Tsolakidou et al., 2019a).

There is a substantial number of studies suggesting that complex inocula can provide plants with increased disease resistance and growth promotion effects as compared to single strains (Rolli et al., 2015; Santhanam et al., 2015; Wei et al., 2015; Molina-Romero et al., 2017; Niu et al., 2017; Berendsen et al., 2018; Tsolakidou et al., 2019a). Bacterial strains that show little or no effects as single inoculants can exhibit plant growth promotion effects when used in a consortium (Raaijmakers and Weller, 1998; Berendsen et al., 2018).

**Figure 2:**
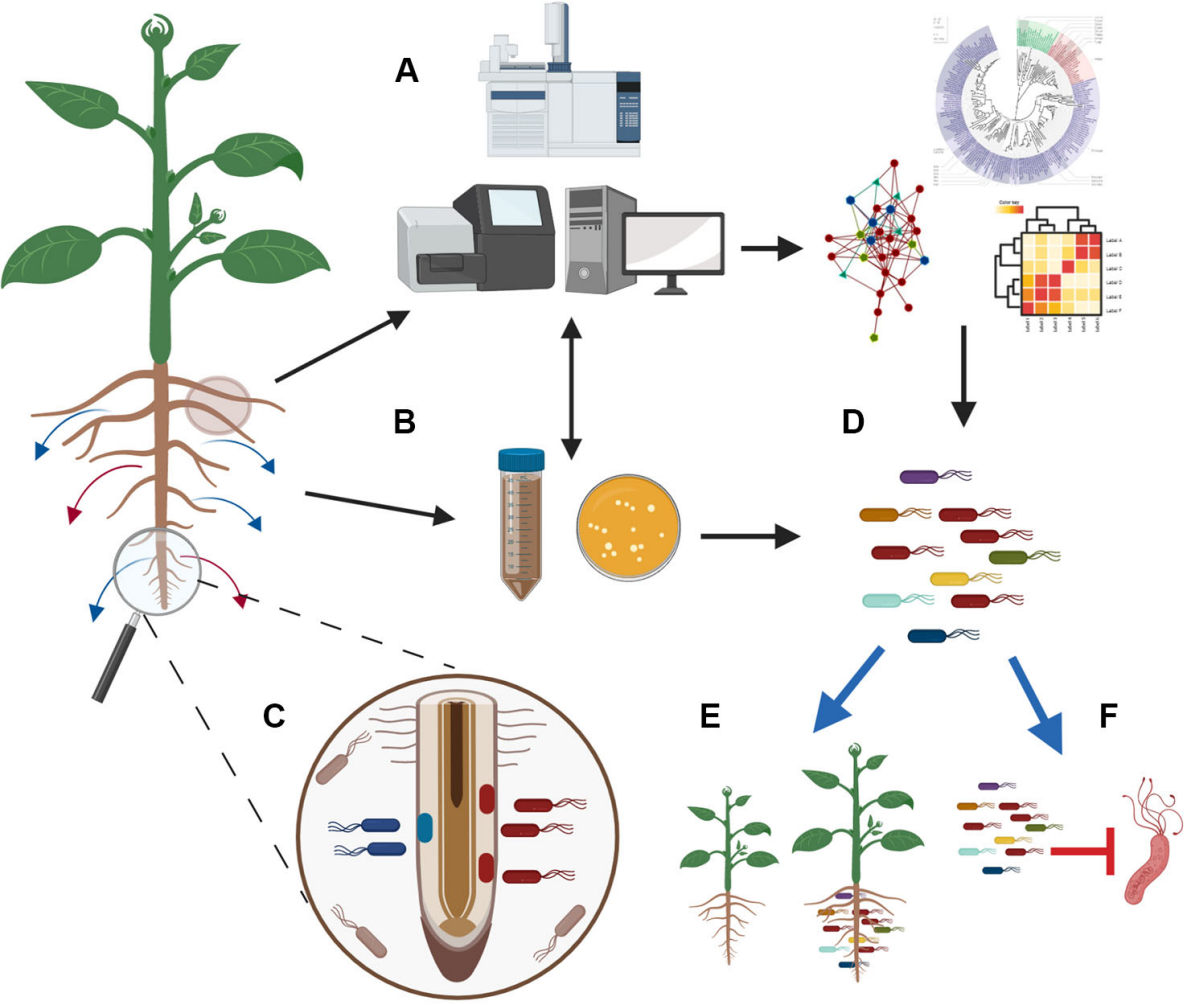
Integration of modern technologies to engineer microbial inoculants that boost plant growth and suppress pathogens. Plants respond to stresses and change their exudation. To unravel how changes in exudation affect microbiome composition and functions, we need to couple advance metabolomic techniques with metagenomics sequencing **(A)** and culture-based methodologies **(B)**. At the same time, there is promise for the use of exometabolomics methodologies and spatial metabolomics that can help in finding where specific exudates are produced and how the microbes around the exudation site are affected **(C)**. Analysis of the generated data in depth will allow the characterization of the microbial communities that respond to exudates and the identification of networks that will reveal how microbes interact and contribute in the microbiome assembly **(A)**. The parallel isolation of a representative fraction of the root microbiome **(B)** will allow to link descriptive data with the isolated microbes and will guide the design of synthetic communities **(D)**. Testing of these synthetic communities with different hosts under different conditions (e.g. biotic/abiotic stress/*in vitro*/in soil/in field) will facilitate the selection of synthetic communities that can promote plant growth **(E)** and suppress pathogens **(F)** in a consistent and reproducible manner. The figure was designed with Biorender (https://biorender.com/).

The prospect of using microbial mixtures as plant inoculants that can positively affect plant properties is an emerging field of research (**Figure 2**). However, the complexity of experimentation is exponentially increasing when using synthetic microbial communities as compared to single strain inoculants. Thus, successful implementation of microbial consortia with desired host outputs will depend on our understanding of how microorganisms interact with one another and with their hosts in natural ecosystems. To this direction, synthetic microbial communities have been widely adopted for fundamental discoveries in plant microbiomes research as a reductionist approach to simplify and especially control each component of this complex system (Bai et al., 2015; Lebeis et al., 2015; Finkel et al., 2019). Indeed, as cleverly postulated by Vorholt and colleagues (2017), the true strength of a synthetic community is that each member of the community can be singularly added or substituted, and this can be even accomplished at a functional level by silencing or expressing specific genes.

However, controlling each member of a large community would bring to a factorial number of possible combinations, making it impossible to control. Recently, Paredes and colleagues (2018) developed a machine learning computational approach to design a bacterial synthetic community. This method was based on the “cry-for-help” theory, consisting in the construction of a neural-network model that received as inputs the growth rate of a pool of bacterial isolates grown with the root exudates of phosphate starved plants, and the phosphate content of shoots of plants in binary interaction with each one of these single bacterial isolates. This method allowed to design a synthetic community with consistent predictable plant phenotypes. In parallel, the construction of the synthetic community based on the “cry-for-help” carried out by Berendsen and colleagues (2018) was more based on a plant-driven approach, where plants effectively attracted a consortium of beneficial bacteria which in turn produced desirable plant phenotypes. These examples show that the identification of microbes that mostly respond to plant stress signals can be used as reliable predictors for the discovery of beneficial microbes.

##### Techniques and Workflows to Harness Plants and Engineer Beneficial Microbiomes

Engineering microbiomes to promote plant fitness and health is an emerging scientific field and an approach holding great promise towards the realization of sustainable future agriculture. However, there are many aspects and technical limitations that need to be considered to effectively exploit this technology. Here, we aim to summarize some of these considerations that are extensively discussed in a recent review by Lawson et al. (2019). First, to unravel mechanisms underlying the interactions between hosts and microbiomes, multiple omics techniques need to be integrated (Jansson and Baker, 2016). Metabolomics, metagenomics, plant transcriptomics, metatranscriptomics, and plant genetics are some of the approaches that combined can disentangle the complex interactions occurring between members of the holobiont. A thorough description of these methodologies are beyond the scope of this review, but some recent focused reviews are available for further reading (Van Dam and Bouwmeester, 2016; Levy et al., 2018; O' Banion et al., 2019; Rodriguez et al., 2019). Here, we report some examples where application of a multi-omics approach revealed how selected plant exudates produced under natural or under stress conditions can affect the colonization of roots by specific microbes. Hu et al. (2018) combined metabolomics and amplicon-based metagenomics analysis on two maize genotypes (wild type and a benzoxazinoids precursor mutant) and revealed how the defense-related benzoxazinoids metabolites structure the bacterial and fungal community of the maize rhizosphere. Stringlis et al. (2018b) also exploited the combination of shotgun metagenomics and metabolomics on an array of Arabidopsis mutants to demonstrate that root exudation of coumarins can shape the rhizosphere microbiome. Similarly, Huang et al. (2019) utilized metabolomics and metagenomics to reveal the effect that root-exuded triterpenes have on microbiota composition of the root. On the track of the work by Berendsen et al. (2018); Yuan et al. (2018) revealed the metabolic drivers of the “legacy effect” by combining metabolomics of the root exudates of infected plants with metagenomics analysis of the rhizospheres of these plants. Furthermore, in an elegant combination of exometabolomics, metagenomics and comparative genomics, Zhalnina et al. (2018) demonstrated how temporal dynamic exudation of root metabolites during different plant developmental stages assembled specific microbial communities and enriched for specific microbial functions. In a next step, we need to link how released plant molecules can affect microbial activity and unearth how plant secretions can define which root niches can be colonized by beneficial microbes while at the same time excluding the pathogenic ones (Jacoby and Kopriva, 2018; Levy et al., 2018).

Furthermore, as the blend of root exudates is strictly dependent on plant genotype, it is expectable that different plants attract different microbes that can produce similar effects on different hosts, due to the redundancy of functions of the microbiome. Considering this, we propose to use desirable microbiome functions as selective markers to identify potential beneficial microbes. By exposing different plant species to the same stress conditions, a comparative metatranscriptomics approach would allow the identification of common functions expressed by microbiomes upon the sensing of stress plant signals. Metatranscriptomics has already been used to highlight the most active members of microbiomes in different plant species or to identify bacterial genes expressed during different Arabidopsis life stages (Turner et al., 2013; Chaparro et al., 2014). To date, only a few metatranscriptomics studies have been conducted, due to the difficulties of mapping metatranscripts to reference genomes and metagenomes. Again, in this case, using synthetic communities composed of whole-genome sequenced members would facilitate this task. Associating these studies with detailed metabolomic analysis of root exudates from stressed plants would then make the integration of multi-omics techniques more and more reliable (**Figure 2**). All together these strategies would produce an incredible amount of data that still need to be interpreted. For this reason, it is necessary to develop bioinformatics techniques that would allow the reduction and summarization of these data. System biology approaches based on correlation networks have been proposed to discover microbial associations where positive and negative correlations can be used to infer possible synergistic or antagonistic interactions (Agler et al., 2016; Poudel et al., 2016; Van Der Heijden and Hartmann, 2016). With this methodology, it is also possible to identify the so-called microbial hub taxa which represent the most interactive nodes in the networks. In this direction, Agler et al. (2016) established a computational method which identified the plant pathogen *Albugo* and the fungus *Dioszegia* as microbial hubs in the microbiome of Arabidopsis phyllosphere. In a further experiment, through the artificial manipulation of the microbiome it was also demonstrated that the microbes identified as the hubs of the network, also represented “keystone taxa” as they drove the composition and function of the microbiome. The concept of “keystone” also has been adopted by Niu et al. (2017) when studying the contribution of individual members of a microbial synthetic community on the rhizosphere of maize plants. In this case, the removal of a singular member caused the collapse of the community functioning with the respective decrease of the richness indexes. These results clearly highlighted that some microbial individuals play a key role in shaping microbial communities on plant hosts.

Another very powerful computational approach is the use of metagenome-wide association study (MWAS). This method derives from the genome-wide association's studies, which rely on the construction of linear mixed models to relate genotypic variations to quantitative observed phenotypes. MWAS have been typically used in human metagenomics studies, i.e. to identify microbial taxa or microbial functions associated with a host phenotypic trait which could be a disease or the host metabolomics profile, by integrating a multi-omics approach (Gilbert et al., 2016). Genome-wide association approach has also been used in the study of plant-microbe interactions, i.e. to identify Arabidopsis loci associated with the ability of plants to maximize benefit from the interaction with the beneficial *Pseudomonas* strain WCS417 (Wintermans et al., 2016). In a plant-microbiome context, Beilsmith and colleagues (2019) propose to use MWAS to find associations between host genes and microbial taxa. MWAS could be very useful to find functional associations between either microbial genes and host genes, or microbial genes and host phenotype, which could also include root exudation profiles.

Finally, to build synthetic microbial communities with consistent beneficial effects for plants in the field, it is essential to understand whether a specific trait of a single strain is expressed in a community level and under multiple contexts (different environmental conditions, hosts, other microorganisms, etc.) (Vannier et al., 2019). This is crucial considering that single strains or synthetic communities that have beneficial effects *in vitro* and under controlled conditions might behave in a different manner in the field. We need also to be aware that the increasing complexity of the synthetic community decreases the feasibility of the large-scale industrial production of microbial inoculants. This should be considered in future plant-microbiome studies with a translational intent, since a number of methodologies and tools need to be combined to design small and effective synthetic communities that can provide the host plants with consistent and predictable outcomes.

## Author Contributions

All authors have contributed to the structure and writing of this review, have read and approved it for publication.

## Funding

The authors gratefully acknowledge MIUR (Ministry for Education, University and Research) for financial support (MIUR- PRIN 2017, grant number PROSPECT 2017JLN833_005).

## Conflict of Interest

The authors declare that the research was conducted in the absence of any commercial or financial relationships that could be construed as a potential conflict of interest.
